# Unleashing of cytotoxic effects of thymoquinone-bovine serum albumin nanoparticles on A549 lung cancer cells

**DOI:** 10.1515/biol-2022-1000

**Published:** 2024-11-26

**Authors:** Bala Baskaran Durga, Vinayagam Ramachandran, Bakthavatchalam Senthil, Vasthi Gnanarani Soloman, Mohamed Soliman Elshikh, Saeedah Musaed Almutairi, Zhi-Hong Wen, Yi-Hao Lo

**Affiliations:** Faculty of Allied Health Sciences, Meenakshi Academy of Higher Education & Research, Chennai, India; Department of Biotechnology, Institute of Biotechnology, College of Life and Applied Sciences, Yeungnam University, 280 Daehak-Ro, Gyeongsan, Gyeongbuk, 38541, Republic of Korea; Department of Chemistry, Faculty of Engineering and Technology, SRM Institute of Science and Technology, Ramapuram, Chennai, Tamil Nadu, India; Department of Botany and Microbiology, College of Science, King Saud University, P.O. 2455, Riyadh, 11451, Saudi Arabia; Department of Marine Biotechnology and Resources, National Sun Yat-sen University, Kaohsiung, 80424, Taiwan; Institute of BioPharmaceutical Sciences, National Sun Yat-sen University, Kaohsiung, 804201, Taiwan; Department of Family Medicine, Zuoying Armed Forces General Hospital, Kaohsiung, 81342, Taiwan; Department of Nursing, Meiho University, Pingtung County 91200, Taiwan

**Keywords:** A549 cells, thymoquinone, bovine serum albumin, cytotoxic, anticancer activity

## Abstract

This research examines the cytotoxic consequences of thymoquinone-loaded bovine serum albumin nanoparticles (TQ-BSA NPs) on the A549 lung cancer cell line. UV-visible (UV–Vis) spectroscopy, Fourier transform infrared spectrophotometer (FT-IR), scanning electron microscopy (SEM), and dynamic light scattering (DLS) were employed to verify the biogenic TQ-BSA NPs’ size, shape, and distribution. UV–Vis spectrophotometry indicated peaks at 200–300 nm, 500–600 nm, and a prominent peak at 700–800 nm, confirming the presence of TQ-BSA NPs. The polydispersity index, as confirmed by DLS, indicated a solvent distribution in water, accompanied by a zeta potential value of 126.2 ± 46.8 mV. The average size of TQ-BSA NPs was confirmed to be 187 ± 8 nm by SEM. TQ-BSA NPs reduce colony formation in the A549 lung cancer cell line in a dose-dependent manner relative to the control group. Protein expression analysis indicated that TQ-BSA NPs promoted programmed cell death by increasing pro-apoptotic levels and decreasing anti-apoptotic levels. TQ-BSA NPs demonstrated inhibition of cancer cell proliferation and promotion of apoptosis and exhibited significant efficacy against cancer cells at low concentrations. As a result, they have the makings of a promising chemotherapeutic agent for low-dose, long-term administration.

## Graphical abstract

**Figure:**
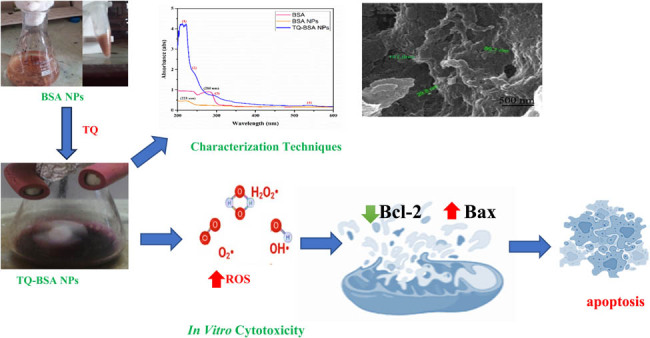

## 1 Introduction

The World Health Organization defines wellness as a multidimensional state encompassing physical and mental well-being devoid of disease. Cancer ranks as the second leading non-communicable disease, following congestive heart failure, and continues to be a significant global issue. Pharmacokinetic challenges, including inadequate biodistribution, incomplete physicochemical characteristics, negative consequences, and a brief circulation half-life, restrict the therapeutic effectiveness of both established and experimental anticancer agents, consequently diminishing drug exposure to tumor cells [1,2]. In India, the most common types of cancer are breast, oral, cervical, lung, stomach, colon, and rectal cancers. Modifiable risk factors for cancer development are primarily influenced by environmental factors associated with lifestyle choices [3]. Research in the medical field emphasizes cancer diagnosis and treatment, along with the long-term health effects of therapeutic interventions [4].

Investigational cancer therapeutics face challenges in targeting specific sites within the body due to suboptimal physicochemical properties. The drugs exhibit side effects and have a short duration of action within the body [5]. These limitations result in impaired biodistribution attributed to unfavorable partitioning coefficients and inefficiencies in passive targeting. The medical field has shifted its focus to nanomedicine to tackle the therapeutic challenges posed by conventional drug delivery methods. This discipline employs the unique physical and chemical characteristics of nanomaterials to design and develop innovative drug distribution platforms, which may transform cancer treatment and address other diseases.

Plants are widely available, less expensive than conventional treatments, and commonly used as herbal remedies for various ailments [6]. Many plants contain antioxidant molecules, and natural antioxidants are becoming more and more popular than manufactured ones [7]. Normal oxygen and exogenous factor metabolism regularly produce reactive oxygen species (ROS) or free radicals [8]. Plant-based medicines continue to provide therapeutic options with fewer side effects, driving significant research interest [9,10]. These chemical processes may cause damage that reduces cell viability *in vitro.*

Albumin is one of the nanomaterials that has attracted significant interest in targeted medicine delivery. Its unique properties, such as drug loading capacity, water solubility, biodegradability, biocompatibility, and ability to transport both lipophilic and hydrophilic drugs, make it especially appealing [11]. Research has linked antioxidant-rich diets to a reduced incidence of chronic diseases, including cancer, neurodegeneration, and diabetes [12]. By aiding in the scavenging of free radicals, antioxidants shield cells from oxidative damage and mitigate the deleterious effects on proteins, lipids, and nucleic acids. Protein nanoparticles’ (NPs’) capacity to traverse physiological barriers and target tumor sites has garnered significant interest in pharmaceutical and nutraceutical research [13].

*Nigella sativa* seeds (*N. sativa*) have many bioactive compounds, such as the main ingredient thymoquinone (TQ) (2-isopropyl-5-methyl-1,4-benzoquinone), as well as monoterpenes like *α*-pinene and *p*-cymene, unique alkaloids like nigellone and nigellimine, and a saponin [14,15]. TQ has garnered considerable research interest owing to its extensive scope of pharmacological activities, such as antioxidant, anti-diabetic, anti-microbial, and hepatoprotective effects [15,16]. The anticancer potential of TQ is particularly well documented in terms of its other pharmacological benefits. Preclinical studies have demonstrated TQ pleiotropic properties, highlighting its role as an antioxidant, immunomodulator, and anticancer agent [17]. Studies have demonstrated that TQ treatment enhances immune function, reduces oxidative stress, and shields healthy cells from damage resulting from these stressors and cancer treatment [18]. Herbs and spices, such as *N. sativa*, exhibit anticancer properties that can effectively target tumor growth.

Bovine serum albumin nanoparticles (BSA NPs) can be produced using several techniques, such as thermal gelation, emulsification, and desolvation, and newer methods like nanospray and NP albumin-bound technology [19]. However, the desolvation method for BSA NP preparation is particularly effective for targeted drug delivery. This study aims to improve the anticancer efficacy of TQ-BSA NPs against the A549 lung cancer cell line.

## 2 Materials and methods

### 2.1 Cells and chemicals

A549 lung cancer cell lines were sourced from the American Type Culture Collection located in Manassas, VA, USA. The Dulbecco Modified Eagle’s Medium, fetal bovine serum, penicillin, and streptomycin were obtained from Hyclone (Logan, USA). Acridine orange/ethidium bromide (AO/EB), methyl thiazolyl diphenyl-tetrazolium bromide (MTT), and polyvinylidene fluoride (PVDF) membrane were obtained from Sigma Aldrich Co., USA. Primary rabbit polyclonal antibodies (Bcl-2, Bax, and Caspase-3) and secondary antibodies were obtained from Abcam, USA. All other chemicals and solvents utilized in this experiment were of analytical grade.

### 2.2 Synthesis of BSA NPs

BSA protein NPs were developed with a slight modification to the desolvation method. A total of 100 mg of BSA was measured and subsequently dissolved in 1.0 mL of a 10 mM NaCl solution at a pH of 7.0, utilizing 0.1 N sodium hydroxide (NaOH). NPs were synthesized by the continuous addition of 5.0 mL of ethanol as a solvent while stirring at 500 rpm at 37°C until the solution exhibited cloudiness. The opaque solution of NPs is stabilized through continuous agitation for 30 min, without the addition of further ethanol. Subsequently, 0.16 mL of 8% glutaraldehyde solution was applied to facilitate particle cross-linking.

#### 2.2.1 Purification of BSA NPs

The NPs were purified via several cycles of differential centrifugation at 20,000 rpm for 8 min. The pellet was re-suspended in a 10 mM NaCl solution, and the supernatant was removed. Ultrasonication was conducted on each re-suspended stage at 10-min intervals [20].

#### 2.2.2 Preparation of TQ-BSA NPs

The extraction of *N. sativa* seeds was performed using ethanol as the solvent, following previously established methods [21]. TQ-loaded NPs are synthesized by dissolving 20 mg of isolated TQ in 0.5 mL of ethanol and diluting it to 1.0 mL with Milli-Q water. Two hundred milligrams of BSA were dissolved in 1 mL of Milli-Q water and subsequently added slowly to the aforementioned solution. The precipitated solution was stirred at 500 rpm for 15 min. A few milliliters of ethanol were added gradually and stirred continuously for 30 min to obtain a clear solution. As a result, absolute ethanol, serving as the desolvating agent, was added gradually while maintaining continuous magnetic stirring at 500 rpm, leading to the prompt formation of a translucent suspension. The addition of 0.16 mL of 8% glutaraldehyde facilitates cross-linking with BSA NPs. To complete the reaction, the solution was maintained at room temperature with constant magnetic stirring at 500 rpm for 18 h. The resulting NP suspension underwent centrifugation for 20 min at 12,000 rpm. The procedure was conducted thrice to eliminate unbound medication, free glutaraldehyde, ethanol, and non-desolvated BSA. At each stage, NPs were reconstituted in approximately 10 mL of deionized water and underwent 5 min of ultrasonication. The NPs were subsequently dehydrated to yield a powder.

### 2.3 Characterization techniques

The morphology of the synthesized NPs was analyzed using scanning electron microscopy (SEM) with a Carl Zeiss scanning electron microscope. The absorption characteristics of the synthesized NPs were analyzed using a Shimadzu UV–Visible spectrophotometer (UV–Vis), with samples scanned over the range of 200–800 nm. The Fourier transform infrared spectrophotometer (FT-IR 8400S, Shimadzu, Tokyo, Japan) was employed to identify the chemical compounds present in synthesized NPs and drug-loaded NPs. The spectra were recorded in the absorption range of 400–4,000 cm⁻¹. The particle size and stability of BSA and drug-loaded NPs were analyzed using a dynamic light scattering (DLS) analyzer and zeta potential measurements with a Zeta-sizer (Malvern Instruments, Southborough, UK), respectively.

### 2.4 Assessment of the anti-carcinogenic effect of TQ-BSA NPs on the A549 lung cancer cell line using the MTT assay

The MTT assay was employed to assess the anti-carcinogenic effect by treating A549 cell lines with TQ-BSA NPs. The samples were combined into a 1 mL stock following filtration. Each of the 96-well plates contained 100 µL of diluted isolated chemical (TQ) along with drug-loaded TQ-BSA NPs. To assess the dose-dependent effects, TQ, TQ-BSA NPs, and cisplatin were evaluated at concentrations ranging from 3.125 to 100 μg/mL. The effectiveness of the samples was assessed by incubating 96-well plates at 36°C with 5% CO_2_, followed by examination after 24 h [22].

### 2.5 Staining with AO/EB

AO/EB staining was employed to detect alterations in apoptosis within cell membranes by examining nuclear changes and the formation of apoptotic bodies [23]. Treated and control cells were seeded in a six-well plate at a density of 3 × 10^4^ cells per well, incubated for 24 h with different drug concentrations, and subsequently stained with a 1:1 acridine orange/ethidium bromide mixture. Cells were rinsed with phosphate-buffered saline (PBS) and analyzed using a 40× fluorescent microscope.

### 2.6 Agarose gel electrophoresis

Agarose gel electrophoresis was employed to distinguish DNA fragments according to their size. The negatively charged DNA migrates through the pores of an agarose gel toward the positively charged end when an electrical current is applied, with smaller fragments moving more quickly than larger fragments. The resulting bands can subsequently be visualized with ultraviolet (UV) light. Agarose gel was prepared and poured onto the gel plate without bubble formation. It was allowed to cool for 20 min, after which the combs were removed, and the cellotapes were uncovered. After mixing 5 μL (100–200 ng) of the DNA sample with the dye and loading it into the well using a pipette or capillary tube, the black negative terminal is connected to the top end of the gel, referred to as the cathode, while the red positive terminal is connected to the bottom end of the gel, known as the anode. Electrophoresis commences upon activating the DC power supply at a voltage of 5 V/cm. Upon the tracking dye (bromophenol blue) advancing 1 cm from the bottom end, the current is deactivated, the power supply is removed, and the gel is subsequently stained with 0.5 μg/mL ethidium bromide in sterile distilled water within a plastic tray for 30–45 min. The gel is rinsed with distilled water and subsequently transferred onto the UV transilluminator. The UV light is activated, allowing for the visualization of DNA bands, and a photograph is captured using an orange filter.

### 2.7 Protein immunoblot analysis

A protease inhibitor (1 mM phenylmethylsulfonyl fluoride) was added to 0.01 M Tris-HCl buffer (pH 7.4) for lysing the cell lines treated with TQ-BSA NPs. Protein concentrations were quantified using the Lowry method, and 50 μg of protein from each sample was subjected to SDS-PAGE electrophoresis on 10% polyacrylamide gels. Proteins were transferred to a PVDF membrane and incubated overnight at 4°C with primary antibodies (Bax, Bcl-2, and caspase 3), followed by incubation with horseradish peroxidase-conjugated secondary antibodies. Protein bands were identified through enhanced chemiluminescence [24].

### 2.8 Flow cytometry analysis

Fluorescence-activated cell sorting (FACS) was employed for cell cycle analysis. The cells were stained using fluorescein isothiocyante (FITC)-Annexin V (BD FACS Aria II BSL-2) to assess cell death at different stages of the cell cycle. Cells were treated with the IC_50_ concentrations of isolated TQ and TQ-BSA NPs (62.15 and 24.56 µg/mL, respectively). After treatment, the cells were collected and resuspended in PBS, and cold ethanol was gradually added to reach a final concentration of 70%. The cells were then incubated in an ice-cold environment for 2 h. Following this, the cells were washed with PBS and resuspended in staining buffer containing 100 µg/mL RNase A, 50 µg/mL propidium iodide, and 0.1% Triton X-100. The suspension was left at 4°C overnight. The fluorescence intensity of the nuclei was measured, and the data were analyzed using flow cytometry software [25].

### 2.9 Statistical analysis

Data were analyzed using SPSS 23 software, employing Duncan’s multiple range test (DMRT) for post hoc comparisons. The results are presented as mean ± standard deviation, with a significance level of *p* < 0.05. All experiments were conducted by three independent investigators to ensure the reliability of the findings.

## 3 Results

### 3.1 UV–Vis spectroscopy of the prepared BSA and TQ-BSA NPs

**Figure 1:**
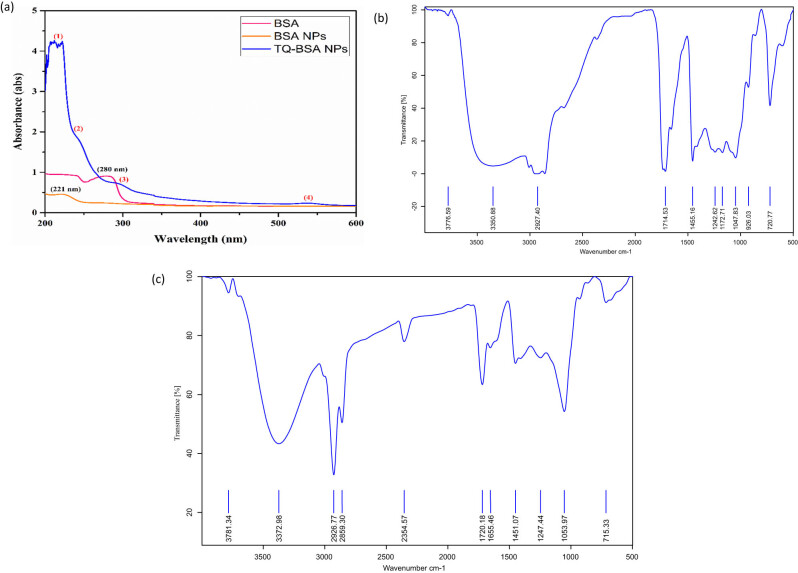
(a) UV–Vis analysis of pure BSA, BSA NPs, and TQ-BSA NPs. (b) FTIR spectra of synthesized BSA nanoparticle and (c) TQ-BSA NPs.

The UV–Vis spectra of pure BSA, BSA NPs, and TQ-BSA NPs are depicted in Figure 1a. The absorption spectra for BSA and BSA NPs were observed within the range of 200 to 800 nm, whereas the TQ-BSA NPs exhibited an extended absorption range, spanning from 200 to 1,000 nm.

### 3.2 FT-IR analysis of synthesized BSA and TQ-BSA NPs

The FTIR spectrum of pure BSA showed characteristic peaks at 3350.88, 2927.40, 1714.53, 1455.16, and 1242.62 cm⁻¹, as displayed in Figure 1b. In contrast, the FTIR spectrum of TQ-BSA NPs exhibited peaks at 3781.34, 2926.77, 1720.18, 1451.07, and 1247.44 cm⁻¹, as shown in Figure 1c and detailed in Table 1. These differences indicate the successful formation of TQ-BSA NPs.

**Table 1:** Characteristic band of BSA NPs and TQ-BSA NPs in FT-IR analysis

Peak value	Bond	Functional group
**BSA NPs**		
3776.59	O–H-stretching	Alcohol
3350.88	NH-stretching	amide A
2927.40	NH-stretching	Amide B
1714.53	C═O stretching	Amide A
1455.16	C–N stretching N–H bending vibration	Amide I
1242.62	C–N stretching N–H bending vibration	Amide II
1172.71	C═O stretching	Amide III
1047.83	C–N stretching	Amides
926.03	═C–H bend	Alkenes
720.77	C═C bending	Alkenes
**TQ-BSA NPs**		
3781.34	O–H stretching	Alcohol
3372.98	NH stretching	Aliphatic 1° amide
2926.77	NH–stretching	Amide a
2859.30	CH stretching	Aldehyde
2354.57	CH stretching	Alkanes
1720.18	C═O stretching vibration	Amide I
1656.46	NH bending	Acids
1451.07	OH bending	Alcohol
1247.44	C–O stretching	Alkyl aryl carrier
1053.97	CO–O–CO stretching	Anhydride
715.33	C–Cl compound	Halo compound

### 3.3 DLS of synthesized BSA and TQ-BSA NPs

**Figure 2:**
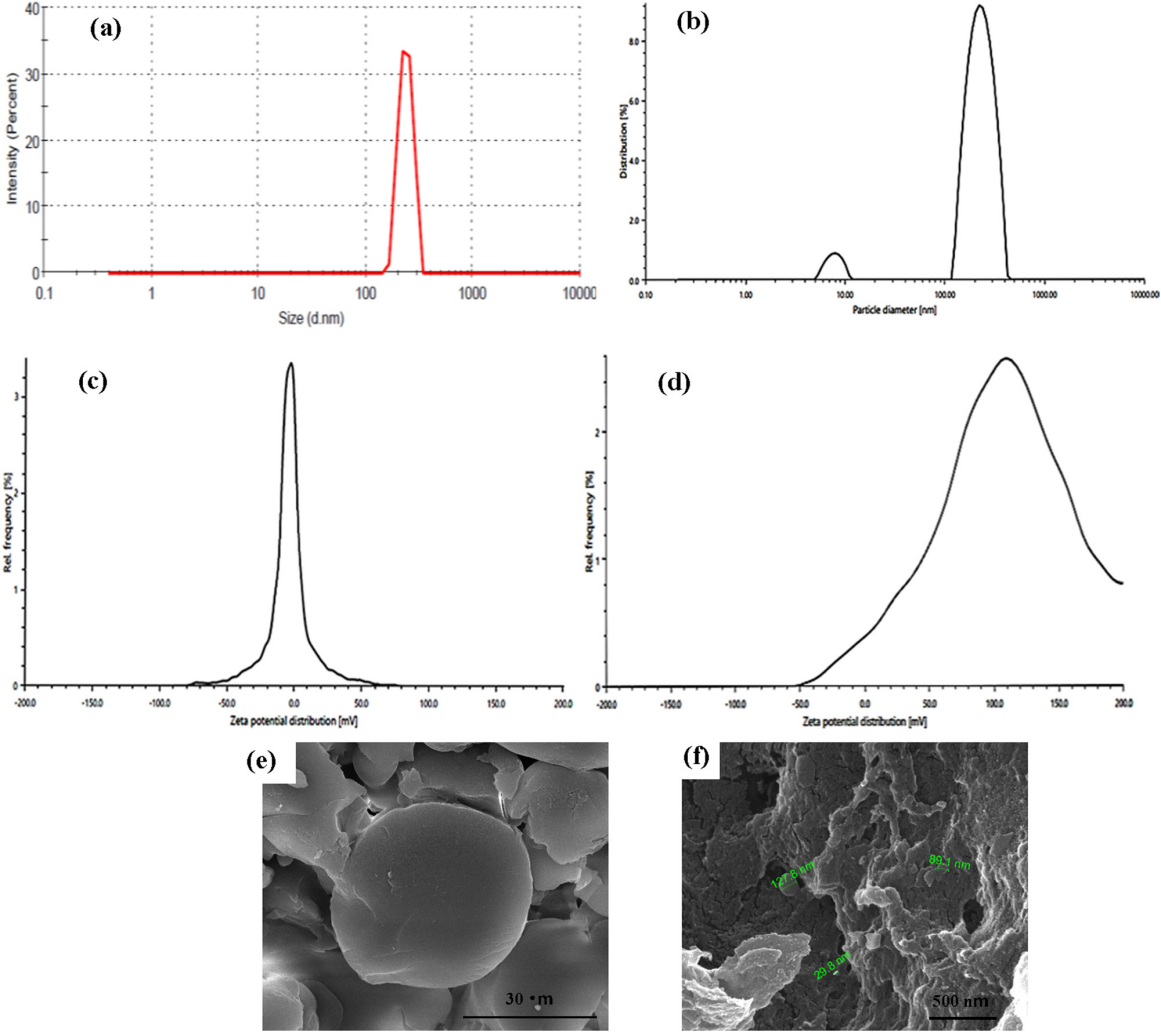
(a) and (b) By performing DLS analysis, the intensity size distribution of BSA NPs and TQ-BSA NPs was identified. (c) and (d) Zeta potential analysis of BSA NPs and TQ-BSA NPs. (e) and (f) SEM analysis of BSA NPs and TQ-BSA NPs.

The DLS measurements of the synthesized NPs showed a single sharp peak representing the size distribution, as illustrated in Figure 2a and b. This indicates a uniform particle size distribution for both BSA and TQ-BSA NPs.

### 3.4 Zeta potential analysis of synthesized BSA and TQ-BSA NPs

The Zeta potential reflects the potential difference between the surrounding layer of dispersed particles and their electric double layer at the sliding plane. As shown in Figure 2c, the BSA NPs exhibited a surface charge of −4.5 ± 1.2 mV when dispersed in water. In contrast, the TQ-BSA NPs, when dispersed in water, displayed a significantly higher zeta potential value of 126.2 ± 46.8 mV, as depicted in Figure 2d. These values indicate the electrostatic stability and surface charge characteristics of the NPs.

### 3.5 SEM analysis of the synthesized BSA and TQ-BSA NPs

SEM images (Figure 2e and f) analysis was performed on the synthesized BSA NPs and the formulated TQ–BSA NPs. The average size of the synthesized BSA NPs is less than 200 nm, while TQ–BSA NPs had an average size of 187 ± 8 nm.

### 3.6 Determine the drug encapsulation efficiency for TQ-BSA NPs

Our experiment investigated a NP-based drug delivery system. We measured how well the NPs (likely made from BSA) bound to the drug molecule (TQ) and how much drug they could carry. The system achieved its highest efficiency (43.2%) with the drug TQ-BSANP.

### 3.7 *In vitro* cytotoxicity assay for TQ, BSA NPs, and TQ-BSA NPs

**Figure 3:**
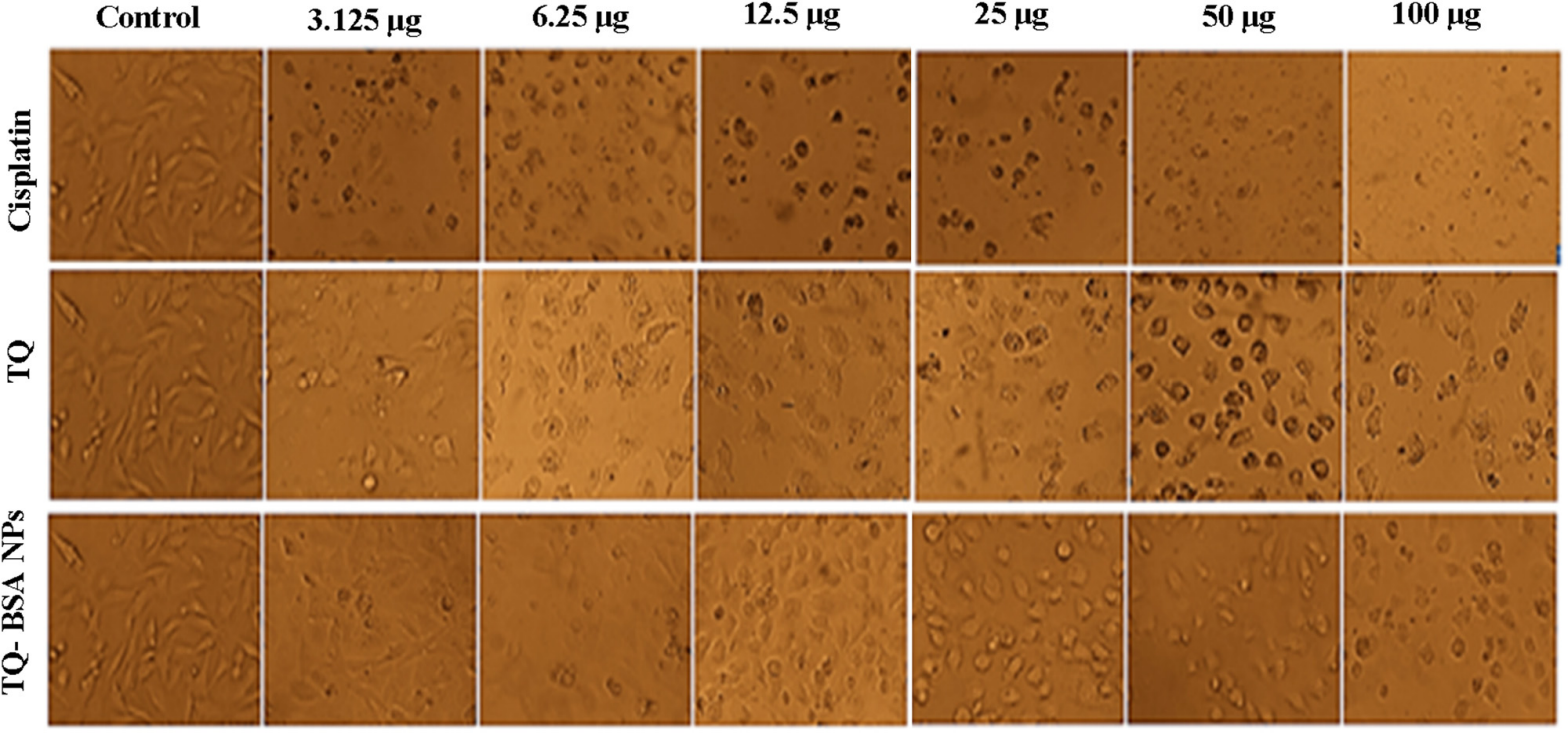
*In vitro* cytotoxicity assay of isolated TQ and TQ-BSA NPs. Microscopic examination at a magnification of 20× was employed to assess cellular morphology and proliferation.

The *in vitro* cytotoxicity of TQ and TQ-BSA NPs was evaluated against the A549 lung adenocarcinoma cell line using the MTT assay to assess cell viability and proliferation. The A549 cancer cells were treated with various concentrations of cisplatin, a commercially used anticancer drug, and incubated for 24 h as a standard comparison. The anti-proliferative activity of isolated TQ, cisplatin, and TQ-BSA NPs was measured after a 24-h incubation, with the results presented in Table 2 and Figure 3. These data allow for the comparison of the cytotoxic efficacy of the NPs against a recognized chemotherapeutic agent.

**Table 2:** *In vitro* cytotoxicity assay of isolated TQ and TQ-BSA Nps

Concentration (μg/mL)	Cytotoxicity (%)		
	Isolated TQ	BSA-TQ NPs	Cisplatin
3.125	2.20 ± 0.17^a^	37.80 ± 0.98^a^	42.05 ± 0.91^a^
6.25	5.75 ± 0.61^b^	43.78 ± 0.97^b^	48.84 ± 0.61^b^
12.50	13.24 ± 0.77^c^	47.98 ± 1.62^c^	60.99 ± 0.55^c^
25.00	24.44 ± 0.75^d^	55.09 ± 1.78^d^	67.77 ± 0.30^d^
50.00	44.47 ± 0.89^e^	60.04 ± 1.08^e^	75.82 ± 0.41^e^
100.00	76.30 ± 0.42^f^	70.06 ± 1.03^f^	96.39 ± 0.15^f^
IC_50_ (μg/mL)	62.15	24.56	2.46
*R* ^2^	0.990	0.892	0.913

Cytotoxicity values are expressed as Mean ± SD (*N* = 3). The data was analyzed by one-way ANOVA test, using IBM SPSS Version 20.0. Mean values within the column followed by different letters are statistically significant (*p* < 0.05) from each other concentration, and the same letters are statistically non-significant (*p* > 0.05) are compared by ANOVA, Duncan’s multiple range test (DMRT), and significant level alpha 0.05.

Visual inspection of the extracted TQ revealed no apparent changes in its chemical composition following treatment, as indicated by minimal to no alterations in concentration. Conversely, the treated cancer cell line displayed dose-dependent morphological alterations, with the highest percentage of cell death observed at increasing dose levels, as depicted in Figure 3. Consistent with free TQ treatment, the TQ-BSA NP-exposed cancer cell line displayed a concentration-dependent response in terms of cellular morphology. Increasing NP concentrations (3.125, 6.25, 12.5, 25, 50, 100 μg/mL) resulted in a statistically significant reduction in the viability of the cell population, as evidenced by a pronounced alteration in cellular morphology in Figure 3. Our investigation revealed that TQ-BSA NPs contributed to an increase in the rate of cell death at every concentration, just as the commercial medication cisplatin. Microscopic examination at a magnification of 20× was employed to assess cellular morphology and proliferation in Figure 3.

The findings demonstrate that a higher dose concentration has superior anti-carcinogenic efficacy, leading to the maximum killing of cancer cells. Present isolated TQ, TQ-BSA NPs, and Cisplatin significantly reduced cell viability as compared to control at 100% cell viability (*p* < 0.05) and significant difference from the concentration using the DMRT statistic (*p* < 0.05). As expected, TQ-BSA NPs exhibit significantly increased cytotoxicity against cancer cell lines, with an IC_50_ of 24.56 µg/mL after a 24-h incubation period. In comparison, the isolated TQ reveals 76.30 ± 0.42% of cell death at a concentration of 100 µg after a 24-h incubation period (IC_50_ = 62.15 µg/mL), while the commercial drug (Cisplatin) treated cancer cell line displays an IC_50_ value of 2.46 µg/mL after a 24 h incubation period in Table 2. TQ, TQ-BSA NPs, and Cisplatin exhibited a significant dose-dependent cytotoxicity on A549 cell (*R* ^2^ = 0.990, 0.892, and 0.913) in Table 2. Previously, Yu *et al.* reported Cisplatin IC_50_ (μg/mL) on A549 2.458 (2.330–2.639) in close agreement with the present study.

### 3.8 Protein expression studies for BSA NPs and TQ-BSA NPs

**Figure 4:**
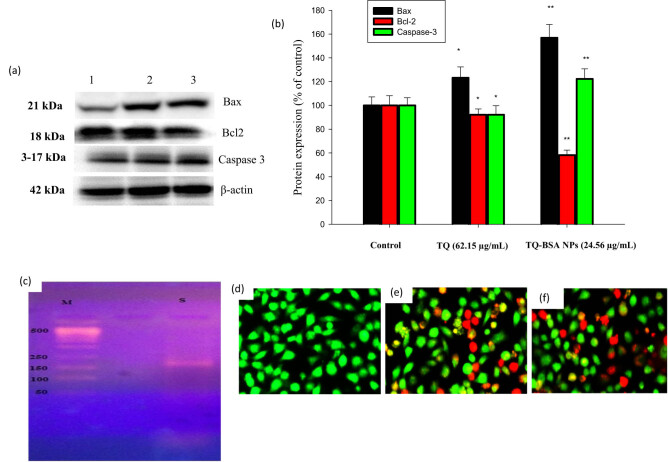
(a) The protein expression changes of Bax, Bcl-2, and caspase-3 in the A549 cell line by Western blot analysis. (b) Densitometric analysis of Bax, Bcl-2, and caspase-3 value were represented as the means ± SD. *p < 0.05 control vs TQ, *p < 0.05 TQ-BSA NPs vs TQ. (c) Lane 1: M – molecular weight of marker of 1 kb ladder, Lane 2: S – sample after 24 h of treatment. (d, e, and f) AO/EB staining A549 cells were labeled by AO/EB 24 h after loading of TQ and TQ-BSA NPs es and were examined under a fluorescent microscope.

The protein expression levels of Bcl-2, Bax, and caspase-3 in A549 cells treated with TQ-BSA NPs are shown in Figure 4a and b. The expression of pro-apoptotic proteins caspase-3 and Bax was progressively increased, while the anti-apoptotic protein Bcl-2 was gradually decreased with treatment at the IC_50_ concentrations of isolated TQ and TQ-BSA NPs (62.15 and 24.56 µg/mL, respectively), as depicted in Figure 4a and b. The effects observed at the IC_50_ concentrations of isolated TQ and TQ-BSA NPs were significantly different compared to the control, indicating the potential of these NPs to induce apoptosis.

### 3.9 Analysis of DNA fragments using agarose gel electrophoresis

As a biological marker for apoptosis, DNA fragmentation analysis was employed biochemically to distinguish between necrotic and apoptotic cells. DNA is taken out of a homogenate of lysed cells and electrophoresed on an agarose gel in this experiment. After subjecting lung adenocarcinoma (A549) cells to BSA loaded with TQ, the cell DNA was extracted and placed onto a 2% agarose gel. The results indicate that there is a connection of some kind between the DNA ladder pattern and the chromatin that was recovered from cancer cells that had been exposed to NPs in Figure 4c.

### 3.10 AO/EB staining

The AO/EB double labeling technique was employed to enhance the detection of apoptotic cell death in cells treated with TQ-BSA NPs. Apoptosis was identified by observing changes in nuclear color. In control (normal) cells, the circular nucleus was uniformly distributed at the center of the cell. Cells treated with the IC_50_ concentrations of isolated TQ and TQ-BSA NPs (62.15 and 24.56 µg/mL, respectively), as determined from the MTT assay, were stained with AO and EB. The late apoptotic cells emitted orange fluorescence, which indicated that they were permeable to both AO and EB. In contrast, green fluorescence was associated with viable cells, as these were only permeable to AO and impermeable to EB. These observations are presented in Figure 4d–f.

### 3.11 Flow cytometer analysis for BSA NPs and TQ-BSA NPs

**Figure 5:**
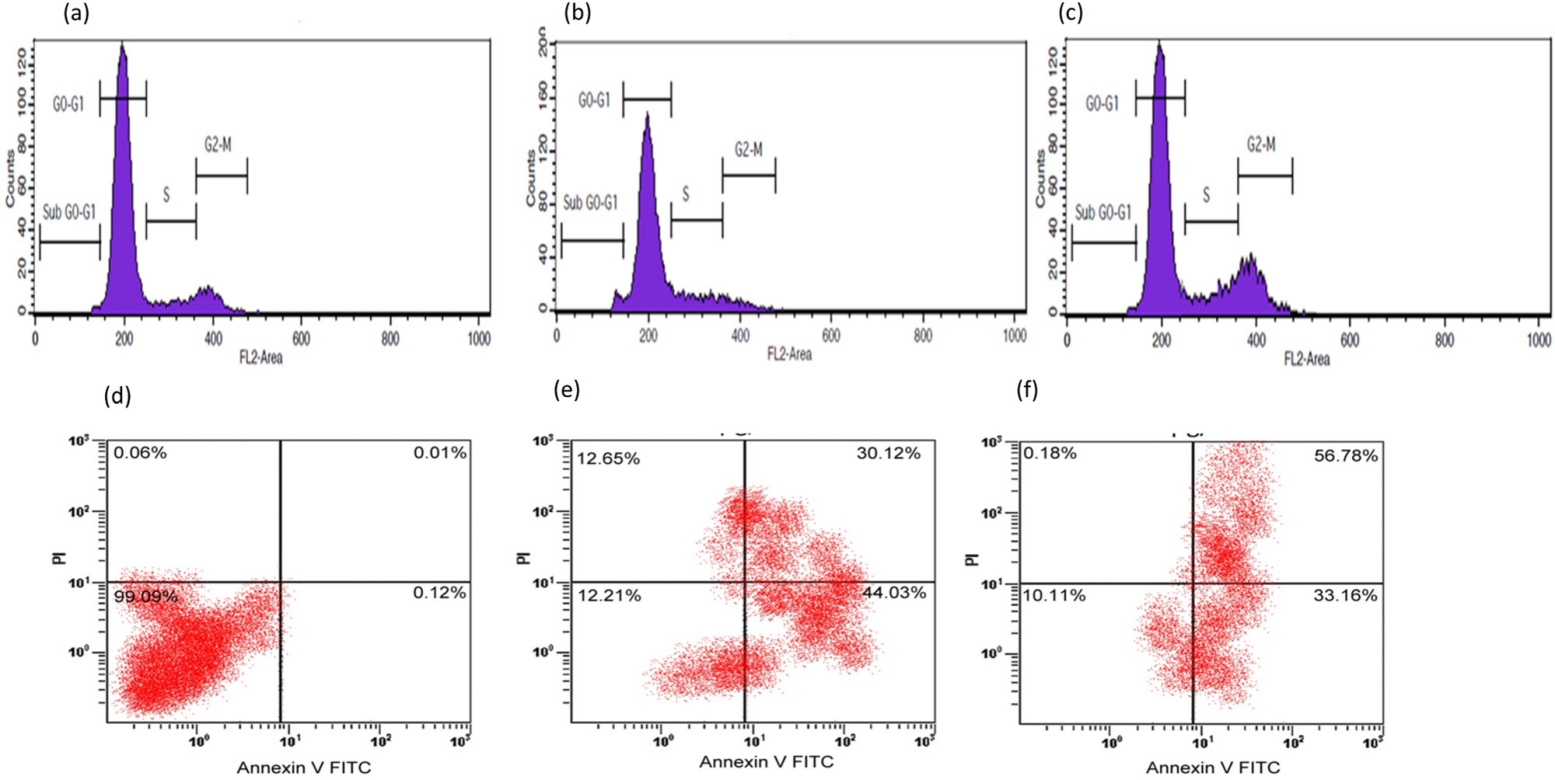
(a)–(c) Flow cytometry analysis of the cytotoxic effect of TQ-BSA NPs in A549 cells. The cells were treated with TQ (62.15 µg/mL) and TQ-BSA NPs (24.56 µg/mL) for 24 h. (d)–(f) the results of the quadrant investigation of fluorescence intensity of A549 cells in Annexin V FITC and PI channels were observed.

Flow cytometry was used to assess the fluorescence intensity corresponding to the uptake of NPs by A549 lung adenocarcinoma cells. The fluorescence intensity of FITC-BSA in each treatment group was quantitatively analyzed based on the number of NPs per vesicle or cell. The data, illustrated in Figure 5a–f, indicated that the relative fluorescence values ranged from 6 to 18, demonstrating a time-dependent increase in NP internalization within the A549 cells. This suggests effective uptake of the NPs over the incubation period.

## 4 Discussion

### 4.1 UV–Vis spectrophotometer

The UV–Vis spectra of Figure 1a of pure BSA peak formed possibly due to the occurrence of amino acid chains, in addition to its feeble absorption because of aromatic amino acids (phenylalanine and tyrosine). BSA NPs peak slightly shifts due to alteration in the protein backbone which substantiates that BSA develops into a nano-sized molecule. It is noteworthy that this brings up the absorption range that is reduced in formulated TQ-BSA NPs where almost five peak changes were observed, and absorption shifts fall between the range of 200–300 nm, 500–600 nm, and major of 700–800 nm in Figure 1a. This clearly states that during nanoparticle synthesis, certain modifications of the backbone of amino acids enhance the interaction of the drug with the aromatic amino acid (phenylalanine and tyrosine) for completion of TQ-BSA NPs.

The results of the study on salicylic acid-loaded protein nanomaterials provide supportive evidence for the findings from UV-Vis spectroscopy, indicating that the drug-binding site on BSA is a lipophilic pocket composed of positively charged surfaces. This configuration readily binds with negatively charged drug molecules, resulting in significant alterations in the absorption spectra of the sample, effectively demonstrating that the drug binds to the protein base for the successful formation of drug-loaded nanoparticles (NPs) [26,27]. Our results have corroborated these findings.

### 4.2 FT-IR analysis

The FT-IR spectrum of pure BSA exhibited characteristic peaks at 3350.88, 2927.40, 1714.53, 1455.16, and 1242.62 cm⁻¹, as shown in Figure 1b. These peaks correspond to the stretching vibrations of OH groups, while amides A and I are associated with NH and C═O stretching vibrations, respectively. Amide II reflects a combination of N–H in-plane bending and C–N stretching vibrations [28]. The synthesis of NPs induces conformational changes that interact with the chemical environment, resulting in slight shifts in the characteristic bonds of the amide functional groups.

The synthesized BSA NPs displayed all the expected prominent spectral peaks, as detailed in Table 1. Key bonding sites include alcohol O–H stretching at 3781.34 cm⁻¹, amide A NH stretching at 2926.77 cm⁻¹, amide I C═O stretching at 1720.18 cm⁻¹, amide II representing the coupling of N–H bending and C–N stretching at 1451.07 cm⁻¹, and amide III corresponding to N–H in-plane bending and C–N stretching at 1247.44 cm⁻¹, as depicted in Figure 1c. Additional peaks observed in the spectra of BSA NPs included NH stretching of aliphatic 1-amines at 3372.98 cm⁻¹, CH stretching from aldehydes at 2859.30 cm⁻¹, CH stretching from alkanes at 2354.57 cm⁻¹, NH bending of acids at 1655.46 cm⁻¹, CO–O–CO stretching at 1053.97 cm⁻¹, and anhydride C–Cl at 715.33 cm⁻¹. Notably, halo compounds were generated only in drug-loaded NPs [29]. These findings confirm the successful synthesis of TQ-BSA NPs, as detailed in Table 1. The results were compared with previous FTIR studies of protein NPs [30,31].

### 4.3 DLS and zeta potential

The size distribution of the prepared NPs, as determined by the DLS method, is shown in Figure 2a and b. The scattering of light by dispersed NPs is proportional to the sixth power of their radii. When the particles are approximately one-tenth the wavelength of the incident light (*λ*/10), the scattered light retains the same energy as the incident light, resulting in elastic scattering that is not angle-dependent (Rayleigh scattering).

The DLS analysis revealed that the prepared BSA NPs exhibited a sharp single peak, indicating their purity and uniform nanosize. Similarly, the drug-loaded BSA NPs also displayed a distinct, sharp peak, confirming their uniformity, purity, and diameter of 187.9 nm. In contrast, the size distribution of the TQ-BSA NPs demonstrated a diameter range within the acceptable limits, along with a polydispersity index (PDI) of less than 0.5%. Previous studies have shown that a low PDI is crucial for ensuring highly monodispersed particles in drug delivery applications [32]. Thus, the DLS results suggest that TQ-BSA NPs are highly effective for cancer treatment.

The zeta potential reflects the potential difference between the layer of dispersed particles surrounding electrophoretically mobile particles and their electric double layer at the sliding plane. Previous research has indicated that the addition of glutaraldehyde and variations in pH can significantly influence the surface charge of NPs, subsequently affecting the electrostatic potential and dispersion stability of BSA NPs in solution [26]. As illustrated in Figure 2c, the zeta potential measurement for BSA NPs showed a surface charge of −4.5 ± 1.2 mV when dispersed in water, which is relatively lower than values reported in previous studies. This reduction in surface charge may be attributed to alterations in the secondary structure of the NPs, leading to decreased surface charges during NP assembly.

Zeta potential serves as an indicator of the colloidal stability of NP suspensions. NPs with high absolute zeta potential values–whether strongly positive or negative–are generally more stable due to the increased electrostatic repulsion between particles, which helps prevent aggregation. In this study, the zeta potential of TQ-BSA NPs was measured at 126.2 ± 46.8 mV (Figure 2d), indicating a strong positive charge. This suggests that the NPs possess good stability and are less prone to aggregation. Such a favorable zeta potential enhances the NPs’ ability to maintain a stable state by inhibiting aggregation [32].

In biological environments, including blood and cell culture media, NPs often interact with a variety of proteins and ions. A high zeta potential can help prevent protein halo formation, aggregation, or destabilization caused by salts or serum proteins, thereby preserving the NPs’ functionality. The strong positive charge of TQ-BSA NPs implies that the formulation is likely to remain stable in biological fluids, enhancing their bioavailability. Therefore, based on various studies, it can be concluded that the synthesized NPs demonstrate excellent stability for drug delivery applications.

Additionally, NP size is a crucial factor influencing cellular uptake. Research indicates that NPs less than 200 nm in size are optimal for internalization by cancer cells, such as the A549 lung cancer cell line. This size range facilitates the exploitation of receptor-mediated endocytosis pathways, which are more efficient for NPs of this dimension. If TQ-BSA NPs fall within this range, they are expected to exhibit higher uptake efficiency, contributing to their therapeutic efficacy. Smaller NPs (<200 nm) also tend to have improved tumor penetration and are more likely to accumulate at tumor sites via the enhanced permeability and retention effect, which is vital for enhancing drug delivery in cancer therapy. A more detailed discussion of the size data obtained from DLS could further elucidate how the size of TQ-BSA NPs might influence their ability to reach and be retained within the tumor microenvironment.

### 4.4 SEM analysis

As shown in Figure 2e and f, SEM analysis was conducted on the synthesized BSA NPs and the formulated TQ-BSA NPs. This analysis confirms that both types of NPs exhibit a similar spherical morphology. Furthermore, it demonstrates that the surfaces of both BSA and TQ-BSA NPs are largely smooth. The average size of the synthesized BSA NPs is less than 200 nm, while the average size of the drug-loaded NPs is 187 ± 8 nm. This finding is consistent with the DLS results, validating that both techniques yield comparable results. The shape and surface morphology of NPs, as observed through SEM, significantly influence their interaction with cells. Spherical particles are generally internalized more efficiently than other shapes, such as rods or discs. If the SEM results confirm that TQ-BSA NPs are spherical, this could enhance their interaction with the A549 cell line, improving their capacity to deliver TQ effectively. The presence of an uneven surface, noted in previous studies, is also linked to increased drug encapsulation efficiency [26,33].

The size and morphology of the NPs directly affect the drug release profile. Smaller NPs typically facilitate faster drug release due to their larger surface area-to-volume ratio, while larger NPs may offer a slower release, potentially allowing for sustained therapeutic effects. According to research findings, if the synthesized nanocarrier is smaller than 200 nm, it can positively interact with tumor tissues, enhancing the permeability and retention effect, which allows the NPs to remain in circulation longer. Given that the TQ-BSA NPs fall within this size range and contribute to a stable system, they are deemed reliable for drug delivery applications.

### 4.5 Anticancer activity

For biomedical applications, it is essential that the synthesized NPs are compatible with living organisms. The *in vitro* cytotoxicity of TQ and TQ-BSA NPs was evaluated against the A549 lung adenocarcinoma cell line using the MTT assay. The MTT results indicated that TQ, TQ-BSA NPs, and cisplatin significantly decreased cell viability compared to the control group, which maintained 100% viability. Similar findings were reported by Ghazy and Hanafy and Salim et al., who assessed the cytotoxicity of Cetuximab and Propolis-loaded serum albumin NPs against various cancer cell lines, including Caco-2, breast, and lung cancer cells [34,35].

The cytotoxicity of TQ-BSA NPs against the A549 cell line was notably significant, revealing a dose-dependent decrease in cell viability with increasing concentrations of TQ, TQ-BSA NPs, and cisplatin. The IC_50_ values were determined as follows: TQ (62.15 µg/mL), TQ-BSA NPs (24.56 µg/mL), and cisplatin (2.46 µg/mL), as detailed in Table 2. The observed suppression of clonogenic potential in treated cells suggests the anti-tumorigenic potential of TQ-BSA NPs. Previous studies have shown similar cytotoxic effects for catechin and epicatechin-BSA NPs and cisplatin against A549 cell lines, supporting the current findings [36,37].

Understanding whether TQ-BSA NPs induce apoptosis through intrinsic (mitochondrial) or extrinsic (death receptor) pathways is critical, as this would provide insights into their mechanisms of action. Without detailing these pathways, the discussion remains speculative. Elucidating the specific apoptotic pathways could reveal whether key proteins such as caspases, Bcl-2, Bax, and cytochrome c are activated during the apoptotic cascade. Mechanistic clarity is essential since different apoptotic pathways can have varied therapeutic implications. For instance, the intrinsic pathway typically involves mitochondrial damage, whereas the extrinsic pathway relies on receptor–ligand interactions [24,38].

Our current findings suggest that high doses of resveratrol-BSA NPs primarily activate a non-caspase-dependent pathway mediated by apoptosis-inducing factor (AIF), followed by a necrotic programmed cell death pathway [39]. Previous *in vitro* and *in vivo* studies have indicated that caspase activation plays a significant role in the cytotoxicity of drug-loaded NPs. While some studies suggest that TQ induces cell death in human colon cancer cells via a caspase-independent mechanism [40], treatments with agents like thyroid substitute-BSA NPs can lead to cytochrome c release and subsequent caspase-3 activation.

Our results demonstrate that treatment with TQ-BSA NPs led to a gradual decrease in Bcl-2 expression, coupled with an increase in Bax and caspase-3 expression, correlating with the IC_50_ concentrations of the NPs (Figure 4a and b). Previous studies have indicated that AIFs can cause cell death through caspase-independent mechanisms [41]. Mitochondrial apoptosis factors contribute to nuclear condensation, triggering cell death through significant chromatin fragmentation [42]. Previous research also supports the notion that TQ and TQ-loaded cubosomes enhance caspase-3 activation in treated cells [43].

Our findings suggest that TQ-BSA NPs induce the translocation of AIF from the mitochondria to the nucleus in A549 cells. The release of AIF protein after treating A549 cells with apoptogenic agents has been shown to concentrate around and partially translocate into the nuclei. Caspase-3 inhibitors can halt chromatin condensation, AIF migration, and DNA fragmentation [44]. Our data indicate that AIF translocation, rather than caspase activation, may primarily drive DNA fragmentation following TQ-BSA NP treatment, as demonstrated by the fact that pan-caspase inhibition prevented treatment-induced apoptotic cell death. These findings align with prior studies exploring the apoptotic pathways mediated by TQ-BSA NPs.

The essential regulatory processes involved in mitochondrial membrane depolarization include the activation of Bax and its subsequent translocation into the mitochondria. For instance, following treatment with macrophage inflammatory protein supernatant, Bax translocates to the mitochondria within 2 h. The upregulation of mitochondrial Bax is associated with a decrease in AIF and cytochrome *c* levels. AIF and cytochrome *c* must be released from the mitochondria for Bax to bind effectively. To our knowledge, this is the first study to investigate the signaling pathway by which TQ-BSA NPs induce apoptosis in A549 cells via a caspase-independent mechanism.

Further research is necessary to fully understand the signaling pathways that lead to TQ-BSA NP-induced apoptotic death in lung cancer A549 cells. TQ may also represent a potential candidate for chemoprevention and chemotherapy, as it can exhibit similar activity at much lower doses than conventional chemotherapy agents. Notably, TQ-BSA NPs showed potent effects on A549 cells. Previous reports indicate that the release of Ca²⁺ from the endoplasmic reticulum plays a crucial role in AIF release from the mitochondria, which is associated with the Bcl family member Bax. Our findings suggest that the apoptosis induced by TQ-BSA NPs in A549 cells may involve AIF release, along with mitochondrial membrane potential changes and modifications in Bax expression [45,46].

Recent insights into the mechanism by which TQ induces cell death in human lung tumors have revealed that TQ typically disrupts signaling through the AKT/phosphatidylinositol 3-kinase and mitogen-activated protein kinase pathways [47]. TQ consistently inhibits the activity of nuclear factor-κB (RelA/p65) and downstream transcription factors, such as AP-1 [48]. It is believed that TQ affects the transcription of genes involved in apoptosis, including cyclins, cyclin-dependent kinases, Bcl-2, Bax, and apoptosis inhibitors [49]. These results align with our findings, suggesting that TQ-BSA NPs promote apoptosis in the A549 cell line via a mitochondrial-mediated apoptotic mechanism through the inhibition of Bcl-2 protein release, which increases mitochondrial membrane permeability.

Investigating the interactions between various pro-apoptotic and anti-apoptotic proteins involved in mitochondrial-mediated apoptosis will be essential for fully elucidating the action mechanism of TQ-BSA NPs on the A549 cell line. The ability of cytotoxic chemotherapy drugs to induce apoptosis and cancer cell death underlies their clinical use [50]. Importantly, this process does not elicit inflammatory responses, as the cells undergo apoptosis. Given the alarming rise in lung cancer incidence, there is an urgent need for revolutionary anticancer therapies that minimize adverse effects on healthy cells [51]. Hence, the potential of BSA NPs to induce apoptosis in invasive breast cancer cell lines was explored. Morphological changes were assessed using inverted phase-contrast microscopy, along with DNA fragmentation tests and AO/EtBr staining after 24 h. The results indicate that BSA NPs can induce apoptosis in breast cancer cells, likely through the production of pro-apoptotic signaling molecules that stimulate cellular death by increasing ROS levels [52].

It is believed that oxidative stress-inducing agents primarily target cancer cells, which exhibit higher ROS levels than normal cells [53]. The findings provide evidence for the pro-oxidant nature of TQ-BSA NPs, as they enhance ROS levels in cancer cells, consistent with previous reports [54,55]. Prior studies have also indicated that Baicalin-loaded folic acid-modified albumin NPs can trigger apoptosis and oxidative stress in MCF-7 cells [56]. The excessive internalization of NPs may lead to dysfunction that accelerates apoptosis [57]. Further investigation is warranted to determine the optimal treatment strategy.

## 5 Conclusion

In summary, TQ-BSA NPs were successfully synthesized and characterized, and their targeted anticancer activity against the A549 lung adenocarcinoma cell line was investigated. Characterization results confirmed the formation of spherical NPs with an average size of 187 ± 8 nm. Anticancer assays demonstrated the effectiveness of TQ-BSA NPs in reducing cell viability in the A549 cell line. Moreover, TQ-BSA NPs induced programmed cell death by promoting pro-apoptotic factors and downregulating anti-apoptotic factors. TQ, the active therapeutic component, exhibited significant efficacy against lung cancer cells when encapsulated in BSA NPs and administered at low doses, indicating its potential as an anticancer agent utilizing BSA as a nanocarrier.

While the *in vitro* findings provide valuable insights, further research is essential to thoroughly investigate the pharmacokinetic properties of these biological agents in preclinical and clinical settings. Such studies will facilitate the development of innovative treatment strategies in oncology, including the formulation of anticancer drugs that minimize adverse effects, even with prolonged use.
